# VSpipe-GUI, an Interactive Graphical User Interface for Virtual Screening and Hit Selection

**DOI:** 10.3390/ijms25042002

**Published:** 2024-02-07

**Authors:** Rashid Hussain, Andrew Scott Hackett, Sandra Álvarez-Carretero, Lydia Tabernero

**Affiliations:** 1 School of Biological Sciences, Faculty of Biology Medicine and Health, University of Manchester, Manchester Academic Health Science Centre, Manchester M13 9PT, UK; rashid.bioinfo@gmail.com (R.H.); andrew.hackett-2@postgrad.manchester.ac.uk (A.S.H.); 2 Bristol Palaeobiology Group, School of Earth Sciences, University of Bristol, Life Sciences Building, Tyndall Avenue, Bristol BS8 1TH, UK

**Keywords:** virtual screening, chemoinformatics, VSpipe, VSpipe-GUI, AutoDock, AutoDock Vina, drug design, docking

## Abstract

Virtual screening of large chemical libraries is essential to support computer-aided drug development, providing a rapid and low-cost approach for further experimental validation. However, existing computational packages are often for specialised users or platform limited. Previously, we developed VSpipe, an open-source semi-automated pipeline for structure-based virtual screening. We have now improved and expanded the initial command-line version into an interactive graphical user interface: VSpipe-GUI, a cross-platform open-source Python toolkit functional in various operating systems (e.g., Linux distributions, Windows, and Mac OS X). The new implementation is more user-friendly and accessible, and considerably faster than the previous version when AutoDock Vina is used for docking. Importantly, we have introduced a new compound selection module (i.e., spatial filtering) that allows filtering of docked compounds based on specified features at the target binding site. We have tested the new VSpipe-GUI on the Hepatitis C Virus NS3 (HCV NS3) protease as the target protein. The pocket-based and interaction-based modes of the spatial filtering module showed efficient and specific selection of ligands from the virtual screening that interact with the HCV NS3 catalytic serine 139.

## 1. Introduction

AutoDock v4.0 (AD4) is a widely used software for screening large chemical libraries from compound databases [1]. The in vogue tools for ligand docking, such as AutoDockTools [1], Raccoon [2], the Autodock.py plugin of PyMOL [3,4], or UCSF DOCK [5], have either a very steep learning curve or require a specific operating system (OS). The more advanced docking tools such as Glide [6], Gold [7], and FlexX [8] require a commercial license for use, and thus are not freely available, unlike the aforementioned resources. To facilitate structure-based virtual screening (VS), we have previously developed VSpipe, a free and open-source command-line pipeline [9]. Although this initial version streamlined the transition between different software packages and new filtering tools, it remains somewhat cumbersome to use. We have now redesigned VSpipe to be launched via an accessible and OS-independent graphical user interface (GUI). 

VSpipe [9] (hereafter referred to as VSpipe-CLI, where CLI stands for command-line interface) is a semi-automated pipeline for virtual screenings that uses AD4 [1], AutoDock Vina (Vina) [10], and Open Babel [11] to perform multiple steps required for structure-based drug design, ranging from the preparation of receptor and ligands to the visualization of results. Lipinski’s “rule of five” [12], ligand efficiency (LE) [13,14], binding efficiency index (BEI), and surface efficiency index (SEI) [15,16] are employed in VSpipe-CLI to aid in the identification and selection of hits for experimental validation. Recently, Onyango et al. [17] used VSpipe-CLI for in silico identification of new anti-SARS-CoV-2 main protease (M^pro^) molecules. Similarly, various studies have used VSpipe-CLI in identifying hit compounds against selected drug targets such as protein tyrosine phosphatases or a fungal methionine synthase [18,19,20,21].

However, VSpipe-CLI requires users to be well versed in a Linux environment and familiar with the CLI-based third-party software mentioned. In order to run VSpipe-CLI successfully, users need to install several dependencies and modify various settings depending on the OS, which can be challenging without a computational background. In addition, users often need to edit the various commands used during the final filtering steps, which can be error prone and lead to a wrong interpretation of the results. To address these shortcomings, we have developed VSpipe-GUI, a new user-interactive graphical interface that includes additional features to improve the VS experience. 

**Figure 1:**
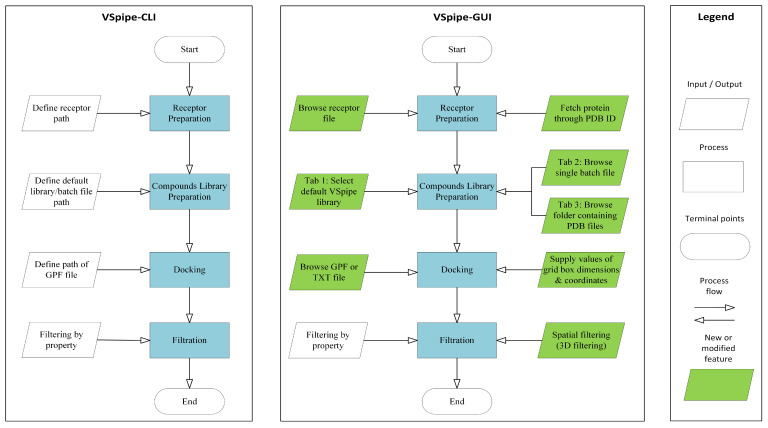
Comparison between VSpipe-CLI and VSpipe-GUI. Blue squares represent the common steps between both versions. Features only available in VSpipe-CLI are represented by uncolored rhomboid shapes. Green rhomboid shapes represent modified/new features implemented in VSpipe-GUI.

VSpipe-GUI integrates different modules that allow users to customize their VS: (i) “Receptor Preparation”, (ii) “Compounds library preparation”, (iii) “Docking”, and (iv) “Filtering”. Briefly, users run a VS through a graphical interface where the options for each of the modules mentioned above can be selected without requiring the command line. Various features have also been included to further improve users’ experience. First, VSpipe-GUI automatically imports protein structures from the Protein Data Bank (PDB) [22] when users enter the corresponding PDB ID. Alternatively, users can use the file browser to upload PDB files with their own generated receptor models. Secondly, the “Compounds library preparation” module in VSpipe-GUI can now prepare library compounds from just individual PDB files. Lastly, a newly developed spatial filtering module has been implemented to filter compounds based on either the binding site they occupy in the target protein, or the desirable interactions with key functional residues. Note that a comparison between the features available in VSpipe-CLI and VSpipe-GUI is illustrated in Figure 1.

The installation process in VSpipe-GUI has been streamlined with fewer dependencies needing installation for launching the tool. Comprehensive and detailed documentation are provided for the installation and running of VSpipe-GUI (https://github.com/rashid-bioinfo/vspipe-gui/tree/master/Installation_Guide accessed on 24 January 2024). Moreover, the implementation of VSpipe-CLI in VSpipe-GUI has been optimized to decrease execution time (more details are given in Section 2.3).

In this study, we tested VSpipe-GUI by screening a fragment compound library against the Hepatitis C Virus (HCV) NS3 protease receptor. We showed that, when compared to VSpipe-CLI, VSpipe-GUI is about 30% faster when using Vina for ligand docking. The NS3/4A protease of the HCV is a critical therapeutic target because it plays a pivotal role in the viral life cycle, making it an attractive target for drug development. Residue serine 139 (S139) is particularly significant as a catalytic residue in NS3/4A. Binding to this residue is essential for blocking protease function and, consequently, for arresting the viral life cycle. HCV infection is as a leading cause of chronic liver diseases with a serious global public health impact. Targeting the NS3/4A protease, especially residues like S139, is thus critical for developing effective treatments [23,24]. We demonstrate the use of the new spatial filtering module in VSpipe-GUI by showing the effective and unbiased selection of fragments binding to the catalytic S139 from the VS on the NS3/4A target. 

## 2. Results

### 2.1. VSpipe-GUI Features

**Figure 2:**
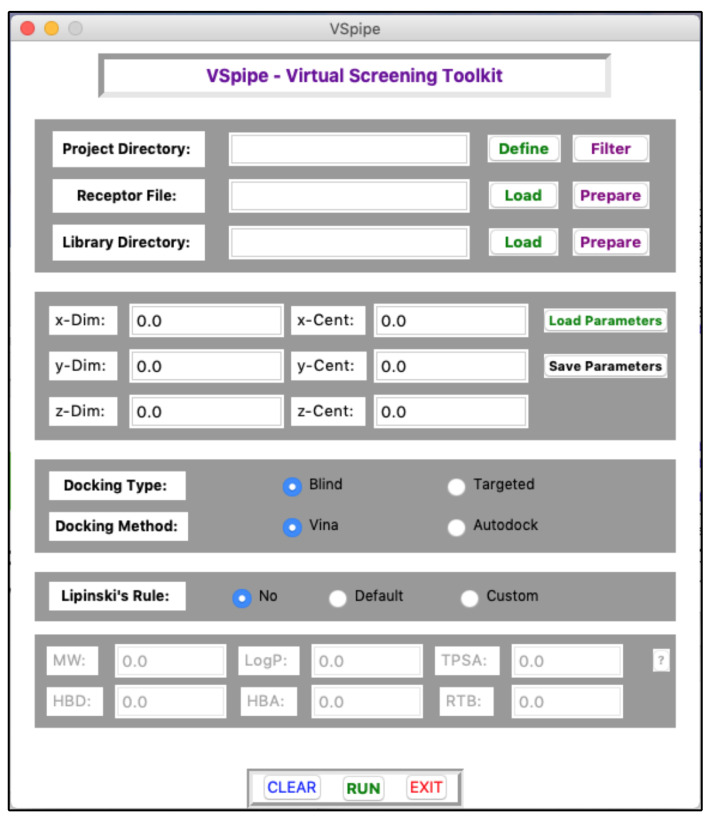
Main interface of VSpipe-GUI where users can customise their virtual screening run.

**Figure 3:**
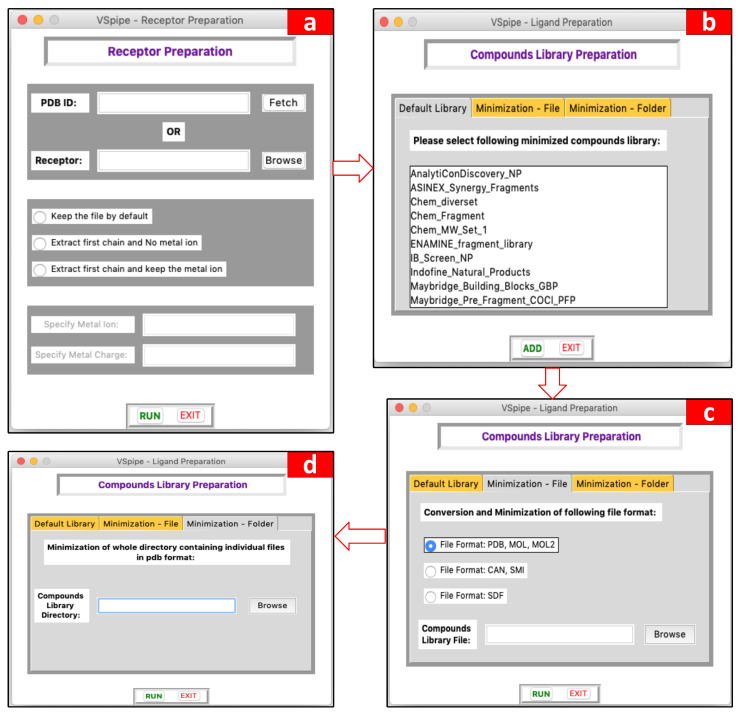
Module options available for users. (**a**) Options for receptor preparation according to users’ needs, (**b**) options to use already minimized compound libraries provided by VSpipe-GUI, (**c**) options to process (and minimize, if not done already) compounds uploaded by users in a single batch file, (**d**) options to process ligand compounds input by users.

Here, we present VSpipe-GUI, a comprehensive graphical interface (Figure 2 and Figure 3) (that implements a redesigned version of VSpipe-CLI where users can interact to customize their structure-based VS according to their target protein and ligand compounds. 

The main GUI window (Figure 2) helps users to specify their preferred project directory for saving results, and for uploading the input receptor target protein and the compound libraries on their local disk. Regarding the Grid Parameter File (GPF), required for docking, users can either manually enter the coordinate values in the main GUI window, or upload a previously saved GPF file in text format. Users may also select the type of docking to take place, either blind or targeted, and the tool to be used for docking, either Vina or AD4. Finally, users are able to choose whether they want to filter the compound library by applying the Lipinski’s rule of five. A more detailed description of the main GUI window is available in Figure S1, and a description of the Lipinski’s rule in Figure S2.

### 2.2. Modules of VSpipe-GUI

#### 2.2.1. Receptor Preparation Module

This module is used to prepare the receptor target protein before starting the VS. Users may either upload their own PDB file for the target protein or specify the PDB ID and allow VSpipe-GUI to retrieve the file directly from the PDB. There are three options to process the PDB file to choose from: (i) extract the first chain of the receptor and clean it from water molecules, metal ions, and other non-protein components; (ii) specify metal ions to keep and the corresponding ion charge if the target protein input is a metalloprotein; or (iii) keep the content of the PDB file intact without changes. The GUI window of this module is shown in Figure 3a (see Figures S3 and S8a for a detailed description of this GUI window and this module workflow, respectively).

#### 2.2.2. Compound Library Preparation Module

This module allows users to prepare ligand compounds prior to the docking step. Users may upload their own compound library in one of the accepted formats (i.e., SDF, PDB, or MOL2), which VSpipe-GUI will then minimize and generate conformers with Open Babel [11]. Alternatively, users may decide to choose one of the eighteen already minimised compound libraries available: AnalytiCon Discovery, Asinex, ChemBridge, ENAMINE, InterBioScreen, Indofine Natural Products, Maybridge, Princeton Natural Products, Specs Natural Products, and Zenobia. The GUI window of this module is described in Figure 3b–d (see Figures S4 and S8b for a detailed description of this GUI window and this module workflow, respectively).

#### 2.2.3. Docking Module

Once the receptor and compound library have been prepared, docking can take place with either AD4 or Vina. First, users define the values of the grid box that will be used to direct the docking onto the receptor (i.e., either generate a GPF file with MGL Tools [1] or reuse previously generated GPF files). Then, users can either upload the resulting GPF file generated or just type the coordinates of the grid box that will be used by VSpipe-GUI to centre the docking site (i.e., the x, y, and z coordinates). In addition, users can choose to further filter the compounds according to the Lipinski’s rule [12]. 

Once all parameters have been specified in this module, users will be able to initiate the docking with the “run” button. Once VSpipe-GUI has successfully run the docking analysis, a CSV file is generated containing compound IDs, their SMILES notations, and the corresponding physic-chemical properties. A new directory will also be created in which the PDB files for the lowest energy compound conformations are saved (see Figure S9a for a detailed description of this module workflow).

#### 2.2.4. Filtering Results by Property Module

**Figure 4:**
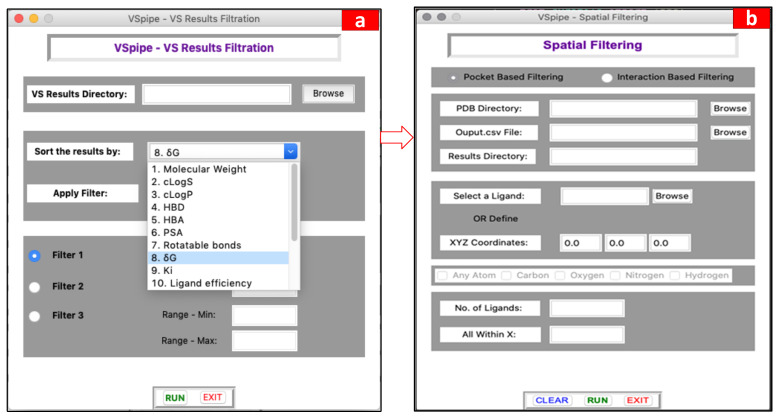
Main GUI windows showing the options that the user can select to summarise the results in the filtering step. (**a**) Options to filter docking results according to various ligand physic-chemical properties, and (**b**) options to constrain the pocket-based and interaction-based spatial filtering (3D).

The output files generated after successfully running a VS include a list of the docked ligands and their corresponding 16 physicochemical properties, and molecular descriptors together with the ligand efficiency indices calculated during the virtual screening (please see the “Materials and Methods” section for details as well as the Supplementary Materials for more information about the workflow). At this stage, users can check these parameters and decide whether to run the filtering module to sort and/or filter the docked ligands by selecting a threshold value for one or more of the properties described. In this way, this module can be run several times with different threshold values for the different properties chosen by the user. Specifically, the parameters to choose from for the filtering are the following: molecular weight (MW), calculated lipophilicity and solubility (cLogS, cLogP), hydrogen bond donors (HBD), hydrogen bond acceptors (HBA), polar surface area (PSA), rotatable bonds, binding affinity (experimental Ki or calculated ΔG), and the ligand efficiency indices, binding efficiency index (BEI), surface-binding efficiency index (SEI), NSEI, NBEI, nBEI, and mBEI. The GUI window of this module is given in Figure 4a (see Figures S5 and S9b for a detailed description of the GUI window and the module workflow, respectively).

#### 2.2.5. New Spatial Filtering (3D Filtering) Module

The aim of the new filtering module is to facilitate the selection of ligands that bind to a specific site, such as the active site or other substrate, or allosteric sites in the target receptor. In VSpipe-CLI, the filtering steps only allowed for the selection of physicochemical parameters but not spatial filtering [9]. Manual evaluation of ligand–protein interactions is a tedious task, which becomes impossible with large libraries. A number of good external options to calculate protein–ligand interactions such as ligGrep and LigPlot [25,26] are available. Nevertheless, we wanted to integrate a similar interaction-based filtering module within VSpipe-GUI to minimise the burden of exporting large numbers of files for use with external software, which reduces the efficiency of the overall VS process. 

The new spatial filtering module allows users to select the docked ligand compounds (i.e., results generated by VSpipe-GUI) based on their binding site interactions with specific target protein residues. This step is important in VS because it enables the selection of ligands based on the known functional relevance of the binding sites or specific residues (i.e., catalytic residues) where clusters are located. In addition, spatial filtering permits the enrichment of the pool of “more likely” active site target inhibitors or other functional binding site inhibitors.

By using this new feature, users can select either a “pocket-based” filtering (i.e., considering interactions between the whole ligand and defined protein site) or an “interaction-based” filtering (i.e., when specific atom types in the ligands are used to define the interactions with atoms or residues in the target). 

In pocket-based filtering, users define a protein-pocket location either using the name of a ligand, which occupies the pocket, or the XYZ coordinates of the centre of the pocket. Next, users specify the size of the pocket in Å and the VSpipe-GUI returns all ligands within the site. Alternatively, users may select few ligands around the centre of the pocket (e.g., return the closest 20 ligands to the centre of the pocket).

In interaction-based filtering, users define a protein residue to select ligands that interact with it by defining the XYZ coordinates of that residue. Subsequently, users may select which atom types in the ligand are involved in the interaction. For example, for a potential hydrogen bond the user may wish to select the oxygen and nitrogen as the ligand interaction atoms. Lastly, users specify the maximum interaction distance desired. 

**Figure 5:**
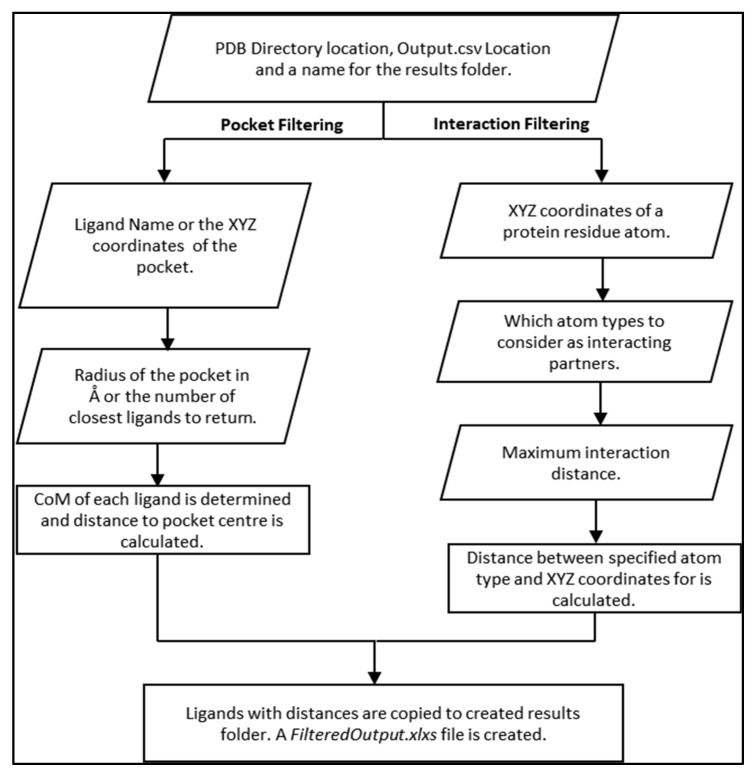
Spatial-based filtering procedure showing the workflow of both pocket-based filtering and interaction-based filtering.

In both cases, once VSpipe-GUI starts, PDB files for both the target protein and the ligand compounds are read, and the coordinates are collected from the input given by the users. The distance to a pocket or protein residue is then calculated, and ligands that match the criteria specified by users are copied to a newly created results directory. An overview of the spatial filtering module and a flowchart describing the operations is shown in Figure 5. The GUI window that users interact with when launching this module is given in Figure 4b (see Figures S6 and S7 for a detailed description of pocket-based filtering and interaction-based filtering processes, respectively).

To demonstrate the application of the new filtering module and its ability to enrich docking results, we used the crystal structure of HCV NS3 protease with the known inhibitor MK-5172 bound in the active site (PDB ID: 5EPN) [27] as our target protein. When running the VSpipe-GUI, we removed the original ligand and water molecules from the target protein prior to running a blind-docking with Vina using the Maybridge 500 fragment library. We then used the spatial filtering module to identify those ligands that were binding to the target protein in a similar fashion to MK-5172.

**Figure 6:**
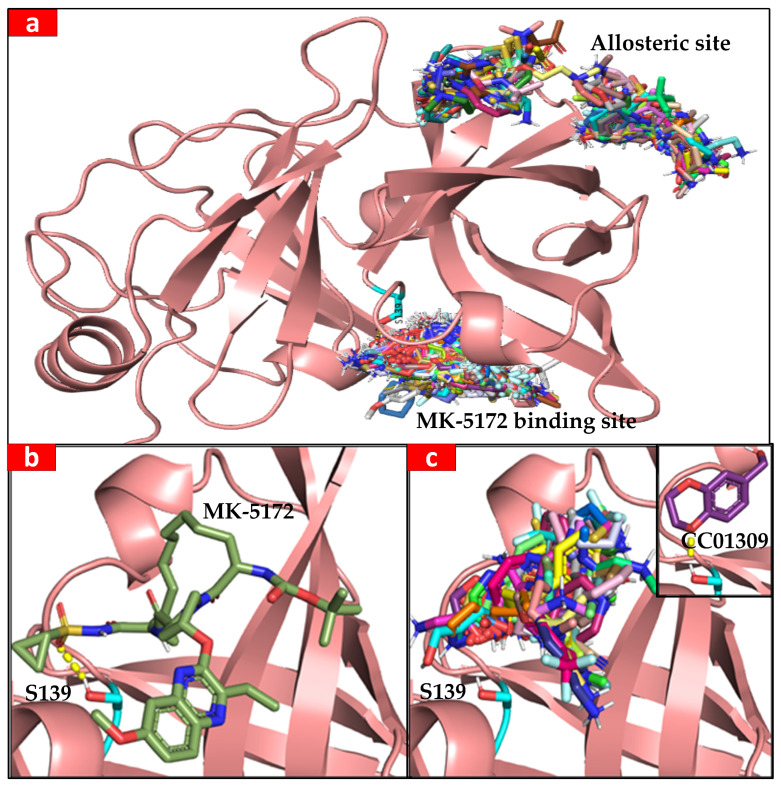
(**a**) General view of MK-5172 and allosteric binding sites in the NS3 protease. The Maybridge 500 fragment library and MK-5712 are docked against the NS3 protease crystal structure; catalytic S139 is highlighted in cyan. (**b**) MK-5712 is bound to the catalytic site of NS3 protease; the hydrogen bond between catalytic S139 (cyan) and the sulfonamide oxygen is shown with a yellow dashed line. (**c**) In total, 93 ligands identified by the pocket-based filtering option occupy the active site. Possible hydrogen bond (shown by the yellow dashes) was identified by the interaction-based filtering option between an oxygen of CC01309 and S139Hg.

The ligand compounds were found to predominantly bind within two distinct sites of the target protein. The first site corresponds to the well-documented active site, where compounds like MK-5712 are known to bind. This site is a recognised target for drug development, essential for the enzymatic function of NS3 [27,28]. The second binding site is located at an allosteric site at the rear of the protein (Figure 6a). While not as extensively studied as the active site, this allosteric site has been previously reported in the scientific literature [29]. Allosteric sites can influence protein function through conformational changes and represent potential targets for therapeutic agents [30,31]. In this case study, we show how VSpipe-GUI allows users to rapidly perform unbiased docking and identify binding sites that have been experimentally validated, as well as new ones for further exploration.

Next, we used the pocket-based filtering option in the spatial filtering module to select only the ligands that were binding to the active site. We selected the coordinates of the active site S139 as the reference for the active site location and set the radius of the pocket to 10 Å. This approach reduced the initial 501 ligands to 82 (Figure 6b). In a second filtering step, we used the interaction-based filtering option to identify those ligands within hydrogen bond distance to the catalytic S139, which interacts with the MK-5712 molecule. 

We selected the hydroxyl hydrogen atom Hγ of S139 as the reference for the target protein and then selected the oxygen and nitrogen atoms as the interaction partners in the ligand, specifying a cut-off distance of 3 Å for the interaction. These criteria identified 14 out the 82 ligands that contained an oxygen or a nitrogen within 3 Å of the target S139OHγ, which greatly reduced the number of ligands for further visual inspection. The interaction-based filtering step also found a possible hydrogen bond between an oxygen of ligand CC01309 and S139Hg of the NS3 protease, similar to that observed in the binding of MK-5712 in the crystal structure [27] (Figure 6c). This approached allowed for the selection of ligands with similar binding modes to that of the MK-5712 inhibitor, indicating a high likelihood for the new ligands to inhibit the target protein [27]. VSpipe-GUI can therefore be used under this scenario to quickly identify a specific binding site by using the new interaction- and pocket-based filtering modules.

### 2.3. Benchmarking the Docking Step between VSpipe-GUI and VSpipe-CLI

We conducted a comparative performance analysis between VSpipe-GUI and VSpipe-CLI to assess the virtual screening efficiency between both versions. Our experiments were conducted using the HCV NS3/4A protease (PDB ID: 5EPN) [27] as the target protein, and we performed virtual screenings with both AD4 and Vina. We ran these analyses on a local machine with Ubuntu 20.04 as its operating system. Table 1 provides an overview of the CPU usage for each scenario.

To expand the initial experiments carried out with the Maybridge library of 98 compounds [9], we analysed a larger compound library containing 500 compounds. This extended the evaluation to assess the scalability and performance of both VSpipe-GUI and VSpipe-CLI versions when dealing with more extensive datasets. Benchmarking both versions with larger libraries allowed us to also explore the capabilities of the spatial filtering module further within VSpipe-GUI. While our initial experiments provided valuable insights into the efficiency of VSpipe-GUI and VSpipe-CLI, the expanded dataset enabled us to assess how well the spatial filtering module accommodates the screening of larger compound libraries and its potential to streamline the compound selection process based on binding site preferences.

Our results show significant efficiency improvements when employing Vina as the docking software in VSpipe-GUI compared to using VSpipe-CLI. These enhancements are summarised as follows:

(1) Detailed improvement in computational time: one of the most significant improvements is the approximately 30% reduction in computational time achieved when running Vina through VSpipe-GUI (please see Table 1 for details). This efficiency improvement can be attributed to an innovative approach that involves batching configuration parameters for all compounds in the library. VSpipe-CLI processes one compound at a time in a sequential approach. In other words, the program reads the configuration parameters for one compound, performs the necessary computations, and then moves on to analyse the next compound. This sequential process requires the program to write and read individual configuration files for each compound. For example, when dealing with a library of 100 compounds, 100 separate configuration files will be generated, thus resulting in a significant time overhead because of the frequent file operations and configuration handling for each compound.

VSpipe-GUI, however, takes a different and more efficient approach than VSpipe-CLI to parameter handling. Instead of processing compounds one at a time in a sequential manner, VSpipe-GUI collects all the configuration parameters for all compounds in the library simultaneously: all the x, y, and z coordinates are collected for every compound at once during runtime. Subsequently, the program applies these parameters to each compound within the library without the need for writing and reading individual configuration files for each compound. VSpipe-GUI effectively batches the configuration parameters by treating them as a single set for the entire library. This streamlined process minimizes the time spent on file operations and configuration handling for each compound, leading to substantial time savings.

(2) Disk space efficiency: beyond the computational time savings, the implementation in VSpipe-GUI also enhances disk space efficiency. By eliminating the need to generate individual configuration files for each compound, VSpipe-GUI significantly reduces disk space usage. This feature proves especially valuable when dealing with extensive compound libraries by simplifying data storage and management, which reduces the overall costs related to such computational resources. Particularly, this feature is advantageous in large-scale virtual screening projects.

(3) Scalability: while initial experiments involved a library of 98 compounds, performance improvements extend to larger compound libraries, including those containing 500 compounds. This scalability feature enables researchers to efficiently apply VSpipe-GUI to high-throughput screening efforts without compromising VS efficiency.

(4) Potential for further optimisation: the observed improvement in computational time primarily results from optimising the configuration parameter handling process. However, it is noteworthy that other features of Vspipe-GUI, such as its accessibility and user-friendly graphical interface, may also contribute to a smoother and more efficient workflow for researchers. Exploring these features and their impact on overall performance may lead to further insights.

## 3. Discussion

We have developed a new interactive virtual screening pipeline, VSpipe-GUI, by implementing VSpipe-CLI [9] with a graphical interface alongside new features. The new version of VSpipe, VSpipe-GUI, has addressed the limitations that users often encountered when using VSpipe-CLI by (i) keeping a simple installation with fewer dependencies, (ii) allowing for an interactive graphical interface instead of using the command line, (iii) enabling users to search their file system to upload input files and easily customise VS options, and (iv) implementing a new spatial-based filtering module. 

Computational docking allows for the processing of large compound libraries by filtering the results to a manageable compound set for subsequent analysis. While VSpipe-CLI could filter docked ligands based on a docking score or on pre-computed physic-chemical properties, the specific location of the ligands or their binding site was not considered. To tackle this problem, we implemented a spatial-filtering module in VSpipe-GUI. Now, users can narrow down the list of final ligand conformations from the VS based on their position in binding sites or specific ligand–protein interactions. In this way, large data sets can now be efficiently filtered by targeting functionalities within the receptor protein such as the active site or known relevant substrate binding or regulatory sites, which has improved the applications of VSpipe-GUI substantially. After testing and validating VSpipe-GUI with the HCV NS3 protease as the target protein, we found that the spatial filtering module could identify ligands interacting with the catalytic S139 residue and specifically compounds with a very similar binding mode to MK-1572, an already reported inhibitor of this protease [32]. 

In conclusion, we have redesigned VSpipe [9] to deliver a user-friendly and interactive tool for VS. VSpipe-GUI offers a simpler implementation and usage, and includes new features that can accelerate the identification of potential hits for experimental validation and drug development.

We believe that the development of user-friendly computational resources and tools such as VSpipe-GUI will help the scientific community by making complex methodologies more accessible to a broader audience. Only by improving the overall VS throughput with more accessible, reproducible, and efficient computational processes will we then be able to enhance the discovery of new compounds for novel and known binding targets of pharmaceutical interest.

## 4. Materials and Methods

### 4.1. Programming Languages

VSpipe-CLI was developed by Álvarez-Carretero et al. [9] in the Bash scripting language as a semi-automated pipeline that combines the usage of AutoDock, Open Babel tools, and in-house Python and R scripts to carry out the VS. Here, we have rewritten this bioinformatics tool as VSpipe-GUI using the Python Tkinter package [33]. 

### 4.2. Comparison of VSpipe-CLI and VSpipe-GUI

VSpipe-CLI and VSpipe-GUI were run separately on a machine running Ubuntu 20.04 LTS [34] with Intel(R) Core ™ i7-5500U CPU @ 2.40 GHz (4CPUs) and 8192 MB of RAM.

### 4.3. Visualization

The software Schrödinger PyMOL version 2.5.4 [4] was used to view the protein–ligand docked poses. 

### 4.4. Target Proteins and Ligands

VSpipe-GUI accepts protein structures in the PDB format [22]. VSpipe-GUI supports both individual files and ligand libraries for small compounds [35]. The formats supported by VSpipe-GUI are PDB, MOL, MOL2, SMI, CAN, and SDF. Current libraries used by VSpipe include those from AnalytiCon Discovery, Asinex, ChemBridge, ENAMINE, InterBioScreen, Indofine Natural Products, Maybridge, Princeton Natural Products, Specs Natural Products, and Zenobia. These libraries are available from various commercial providers and can be pre-formatted for use by VSpipe.

### 4.5. Filtering the Results after the VS

After filtering, VSpipe-GUI creates a new directory with the filtered results: (i) output summary files with the information of the filtered ligands according to the criteria specified by users; (ii) a subdirectory with the PDB ligand files that meet the filtering requirements; and (iii) the ligand efficiency plots corresponding to the filtered ligands. The output summary plots are (a) HBA vs. number of compounds; (b) log P vs. number of compounds; (c) MW vs. number of compounds; (d) NSEI (−log10Ki/NPOL) vs. nBEI (−log10[(Ki/NHEA)]); (e) PSA-number of compounds; and (f) SEI (p(Ki)/(PSA/100 Å2) vs. BEI (p(Ki)/MW(kDa)). Please note that this module is very convenient for users that want to sort and order the screened ligands according to a specific property or various properties. This feature becomes even more useful when thousands of compounds have been screened and users want to narrow down the list of selected compounds to those fitting specific criteria.

### 4.6. Spatial Filtering

The spatial filtering module contains two different filtering options: a pocket-based filtering mode and an interaction-based filtering mode (Figure 4b). When enabling the pocket-based mode, users define the location of a binding pocket using either the XYZ coordinates of an atom in the receptor protein or a specific ligand name bound in the site. Then, users can define a distance range from the specified receptor atom (e.g., 20 Å from oxygen in a catalytic serine residue) or the number of the closest ligands to that receptor atom (e.g., 20 top ligands). In the first instance, VSpipe-GUI calculates the centre of mass (CoM) for each ligand, and then calculates the distance from the specified receptor atom to the computed CoM. VSpipe-GUI then selects those ligands that are located within the distance range specified by users and copies the selected ligand PDB files to the new results directory. In addition, the filtering module generates a new FilteredOutput.xlsx file with the parameters of each selected ligand. In the other instance, when a specific ligand (i.e., reference ligand) is chosen, VSpipe-GUI uses the CoM of that ligand to rank the rest of the ligand conformations according to their distance to the reference. Once this is done, the program will select the top number of ligands pre-defined by users (e.g., 20) and copy the corresponding PDB files to the new results directory, where a new FilteredOutput.xlsx file will be saved. When the interaction-based filtering option is enabled, users define the XYZ coordinates of an atom within the receptor protein residue. Afterwards, users select specific atom types in the ligand (i.e., O, N, C, H) and then the distance range between the protein atom and the ligand atoms (Figure 4b). Vspipe-GUI will then calculate the distance between each specified ligand atom type and the receptor atom. Based on such results, the program will select those ligands within the defined distance criteria and copy the corresponding PDB files into the results directory, where the FilteredOutput.xlsx file will also be saved.

### 4.7. Docking Parameters for HCV NS3 Protease

HCV NS3/4A protease (PDB ID: 5EPN) [27] was used as a receptor. The grid centre for the S139 in the catalytic site was x = −8.063, y = −21.772, z = 13.487, while the grid size was defined to be x = 40, y = 40, z = 40. The grid spacing was set to 0.375 Å. The Maybridge 500 fragment library was used for spatial filtering. Both the Maybridge 98 fragment library and the 500 compound library aforementioned were used for benchmarking Vspipe-GUI and Vspipe-CLI.

## Figures and Tables

**Table 1:** Benchmarking of the docking step when running Vspipe-CLI and Vspipe-GUI with both AD4 and Vina during the screening of two libraries of 98 and 500 compounds, respectively, onto the HCV NS3 protease as a target protein. We ran Vina and AD4 with VSpipe-CLI and VSpipe-GUI on a PC with 8192 MB RAM and Intel(R) Core(TM) i7-5500 CPU @ 2.40 GHz (4 CPUs).

Docking Software	Docking Time with VSpipe-CLI (hh:mm:ss)	Docking Time with VSpipe-GUI (hh:mm:ss)
	**98 compounds**	
Vina 1.1.2	00:14:00	00:09:32
AD4 4.2.6	01:16:26	01:23:10
	**500 compounds**	
Vina 1.1.2	01:28:26	00:49:18
AD4 4.2.6	09:40:41	09:40:17

## Data Availability

VSpipe-GUI can be freely accessed and downloaded from GitHub (https://github.com/rashid-bioinfo/vspipe-gui (accessed on 24 January 2024)). Within this repository, users are provided with an extensive installation guide to set up VSpipe-GUI on Linux distributions (tested on Ubuntu 20.04 but expected to work on other distributions too), Windows (from version 10 and on the Windows Linux Subsystem), and Mac OS X (tested on High Sierra v10.13.6 but expected to work on other latest versions). Step-by-step tutorials are also available to help users run their VS analysis.

## Supplementary Materials

The following supporting information can be downloaded at: https://www.mdpi.com/article/10.3390/ijms25042002/s1.

## References

[1] Morris G.M., Huey R., Lindstrom W., Sanner M.F., Belew R.K., Goodsell D.S., Olson A.J. (2009). AutoDock4 and AutoDockTools4: Automated Docking with Selective Receptor Flexibility. J. Comput. Chem..

[2] Forli S., Huey R., Pique M.E., Sanner M.F., Goodsell D.S., Olson A.J. (2016). Computational protein-ligand docking and virtual drug screening with the AutoDock suite. Nat. Protoc..

[3] Seeliger D., de Groot B.L. (2010). Ligand Docking and Binding Site Analysis with PyMOL and Autodock/Vina. J. Comput. Aided. Mol. Des..

[4] Schrödinger L., DeLano W. (2020). PyMOL. http://www.pymol.org/pymol.

[5] Allen W.J., Balius T.E., Mukherjee S., Brozell S.R., Moustakas D.T., Lang P.T., Case D.A., Kuntz I.D., Rizzo R.C. (2015). DOCK 6: Impact of New Features and Current Docking Performance. J. Comput. Chem..

[6] Friesner R.A., Banks J.L., Murphy R.B., Halgren T.A., Klicic J.J., Mainz D.T., Repasky M.P., Knoll E.H., Shelley M., Perry J.K. (2004). Glide: A New Approach for Rapid, Accurate Docking and Scoring. 1. Method and Assessment of Docking Accuracy. J. Med. Chem..

[7] Verdonk M.L., Cole J.C., Hartshorn M.J., Murray C.W., Taylor R.D. (2003). Improved Protein-Ligand Docking Using GOLD. Proteins Struct. Funct. Bioinform..

[8] Kramer B., Rarey M., Lengauer T. (1999). Evaluation of the FLEXX Incremental Construction Algorithm for Protein—Ligand Docking. Proteins Struct. Funct. Bioinform..

[9] Álvarez-Carretero S., Pavlopoulou N., Adams J., Gilsenan J., Tabernero L. (2018). VSpipe, an Integrated Resource for Virtual Screening and Hit Selection: Applications to Protein Tyrosine Phospahatase Inhibition. Molecules.

[10] Trott O., Olson A.J. (2010). AutoDock Vina: Improving the Speed and Accuracy of Docking with a New Scoring Function, Efficient Optimization, and Multithreading. J. Comput. Chem..

[11] O’Boyle N.M., Banck M., James C.A., Morley C., Vandermeersch T., Hutchison G.R. (2011). Open Babel: An Open Chemical Toolbox. J. Cheminform..

[12] Lipinski C.A. (2000). Drug-like Properties and the Causes of Poor Solubility and Poor Permeability. J. Pharmacol. Toxicol. Methods.

[13] Hopkins A.L., Groom C.R., Alex A. (2004). Ligand Efficiency: A Useful Metric for Lead Selection. Drug Discov. Today.

[14] Hopkins A.L., Keserü G.M., Leeson P.D., Rees D.C., Reynolds C.H. (2014). The Role of Ligand Efficiency Metrics in Drug Discovery. Nat. Rev. Drug Discov..

[15] Abad-Zapatero C., Metz J.T. (2005). Ligand Efficiency Indices as Guideposts for Drug Discovery. Drug Discov. Today.

[16] Abad-Zapatero C. (2007). Ligand Efficiency Indices for Effective Drug Discovery. Expert Opin. Drug Discov..

[17] Onyango H., Odhiambo P., Angwenyi D., Okoth P. (2022). In Silico Identification of New Anti-SARS-CoV-2 Main Protease (Mpro) Molecules with Pharmacokinetic Properties from Natural Sources Using Molecular Dynamics (MD) Simulations and Hierarchical Virtual Screening. J. Trop. Med..

[18] Scott J., Sueiro-Olivares M., Thornton B.P., Owens R.A., Muhamadali H., Fortune-Grant R., Thomson D., Thomas R., Hollywood K., Doyle S. (2020). Targeting Methionine Synthase in a Fungal Pathogen Causes a Metabolic Imbalance That Impacts Cell Energetics, Growth, and Virulence. mBio.

[19] Adams J., Thornton B.P., Tabernero L. (2021). A New Paradigm for KIM-PTP Drug Discovery: Identification of Allosteric Sites with Potential for Selective Inhibition Using Virtual Screening and LEI Analysis. Int. J. Mol. Sci..

[20] Thornton B.P., Johns A., Al-Shidhani R., Álvarez-Carretero S., Storer I.S.R., Bromley M.J., Tabernero L. (2019). Identification of Functional and Druggable Sites in Aspergillus Fumigatus Essential Phosphatases by Virtual Screening. Int. J. Mol. Sci..

[21] Xing Y., Yang R., Yang H., Jiang D., Xu L., Feng L. (2022). Investigation of the Potential Mechanism of the Shugan Xiaozhi Decoction for the Treatment of Nonalcoholic Fatty Liver Disease Based on Network Pharmacology and Molecular Docking. PeerJ.

[22] Sussman J.L., Lin D., Jiang J., Manning N.O., Prilusky J., Ritter O., Abola E.E. (1998). Protein Data Bank (PDB): Database of Three-Dimensional Structural Information of Biological Macromolecules. Acta Crystallogr. Sect. D Struct. Biol..

[23] Romano K.P., Ali A., Aydin C., Soumana D., Özen A., Deveau L.M., Silver C., Cao H., Newton A., Petropoulos C.J. (2012). The Molecular Basis of Drug Resistance against Hepatitis C Virus NS3/4A Protease Inhibitors. PLoS Pathog..

[24] Ashraf M.U., Iman K., Khalid M.F., Salman H.M., Shafi T., Rafi M., Javaid N., Hussain R., Ahmad F., Shahzad-Ul-Hussan S. (2019). Evolution of Efficacious Pangenotypic Hepatitis C Virus Therapies. Med. Res. Rev..

[25] Ha E.J., Lwin C.T., Durrant J.D. (2020). LigGrep: A Tool for Filtering Docked Poses to Improve Virtual-Screening Hit Rates. J. Cheminform..

[26] Laskowski R.A., Swindells M.B. (2011). LigPlot+: Multiple Ligand—Protein Interaction Diagrams for Drug Discovery. J. Chem. Inf. Model..

[27] Soumana D.I., Kurt Yilmaz N., Prachanronarong K.L., Aydin C., Ali A., Schiffer C.A. (2016). Structural and Thermodynamic Effects of Macrocyclization in HCV NS3/4A Inhibitor MK-5172. ACS Chem. Biol..

[28] Summa V., Ludmerer S.W., McCauley J.A., Fandozzi C., Burlein C., Claudio G., Coleman P.J., DiMuzio J.M., Ferrara M., Di Filippo M. (2012). MK-5172, a Selective Inhibitor of Hepatitis C Virus NS3/4a Protease with Broad Activity across Genotypes and Resistant Variants. Antimicrob. Agents Chemother..

[29] Saalau-Bethell S.M., Woodhead A.J., Chessari G., Carr M.G., Coyle J., Graham B., Hiscock S.D., Murray C.W., Pathuri P., Rich S.J. (2012). Discovery of an Allosteric Mechanism for the Regulation of HCV NS3 Protein Function. Nat. Chem. Biol..

[30] Abian O., Vega S., Sancho J., Velazquez-Campoy A. (2013). Allosteric Inhibitors of the NS3 Protease from the Hepatitis C Virus. PLoS ONE.

[31] Xue W., Yang Y., Wang X., Liu H., Yao X. (2014). Computational Study on the Inhibitor Binding Mode and Allosteric Regulation Mechanism in Hepatitis C Virus NS3/4A Protein. PLoS ONE.

[32] Xue W., Ban Y., Liu H., Yao X. (2014). Computational Study on the Drug Resistance Mechanism against HCV NS3/4A Protease Inhibitors Vaniprevir and MK-5172 by the Combination Use of Molecular Dynamics Simulation, Residue Interaction Network, and Substrate Envelope Analysis. J. Chem. Inf. Model..

[33] Grayson J.E. (2000). Python and Tkinter Programming.

[34] Sobell M.G. (2011). A Practical Guide to Ubuntu Linux.

[35] Bourne P.E., Weissig H. (2005). Structural Bioinformatics.

