# Supplementary material for: VSpipe-GUI, an Interactive Graphical User Interface for Virtual Screening and Hit Selection
Source: Int J Mol Sci. 2024 Feb 7;25(4):2002. doi: 10.3390/ijms25042002 (PMC10889202; doi:10.3390/ijms25042002)
Supplement: Supplementary file 1 [file ijms-25-02002-s001.zip › ijms-2814389-supplementary.pdf]

SUPPLEMENTARY MATERIAL

VSpipe-GUI, an Interactive Graphical User Interface for Virtual Screening and Hit Selection

Rashid Hussain [1], Andrew Scott Hackett [1], Sandra Álvarez-Carretero [2,*] and Lydia Tabernero [1,*]

[1] School of Biological Sciences, Faculty of Biology Medicine and Health, University of Manchester, Manchester Academic Health Science Centre, Manchester M13 9PT, UK.

[2] Bristol Palaeobiology Group, School of Earth Sciences, University of Bristol, Life Sciences Building, Tyndall Avenue, Bristol BS8 1TH, UK.

* Correspondence: sandra.ac93@gmail.com; lydia.tabernero@manchester.ac.uk.

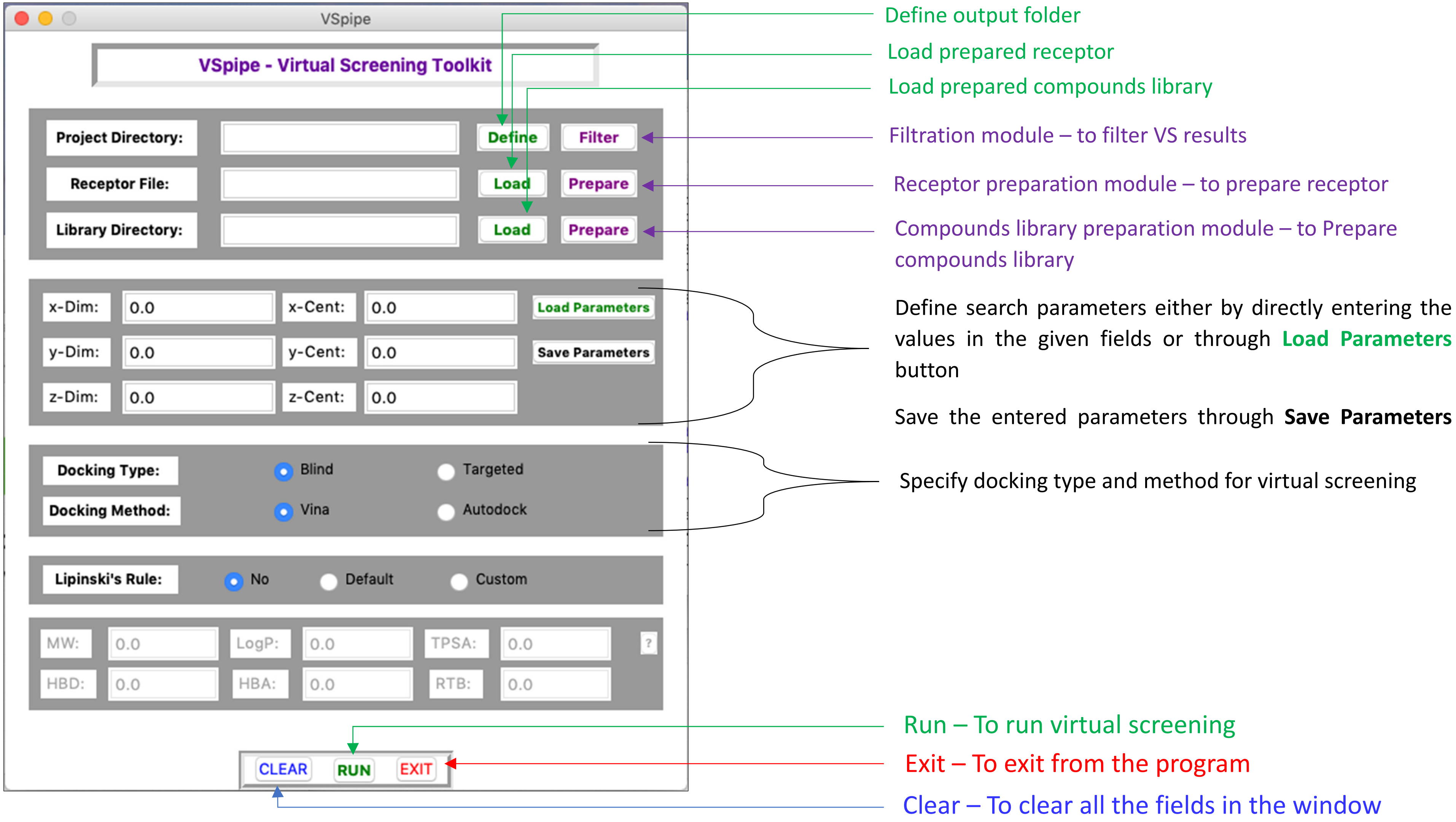

Figure S1. Overview of Main GUI Window

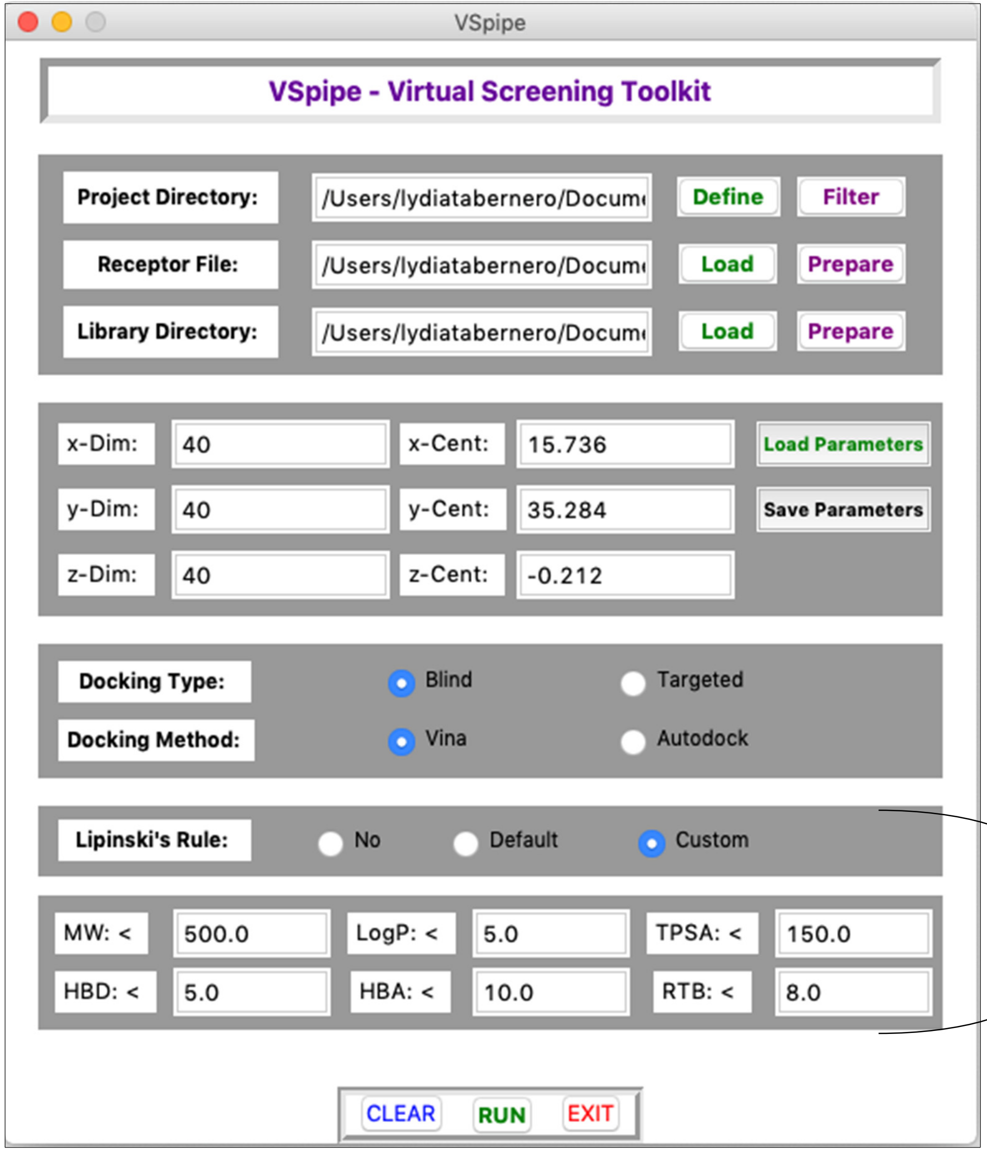

Define physico-chemical parameters to filter the compounds library further based of Lipinski's rule of five.

No – No filter is applied.

Default – Default parameters are applied defined by Lipinski.

Custom – User-defined parameters.

Figure S2. Lipinski's rule of five GUI Window

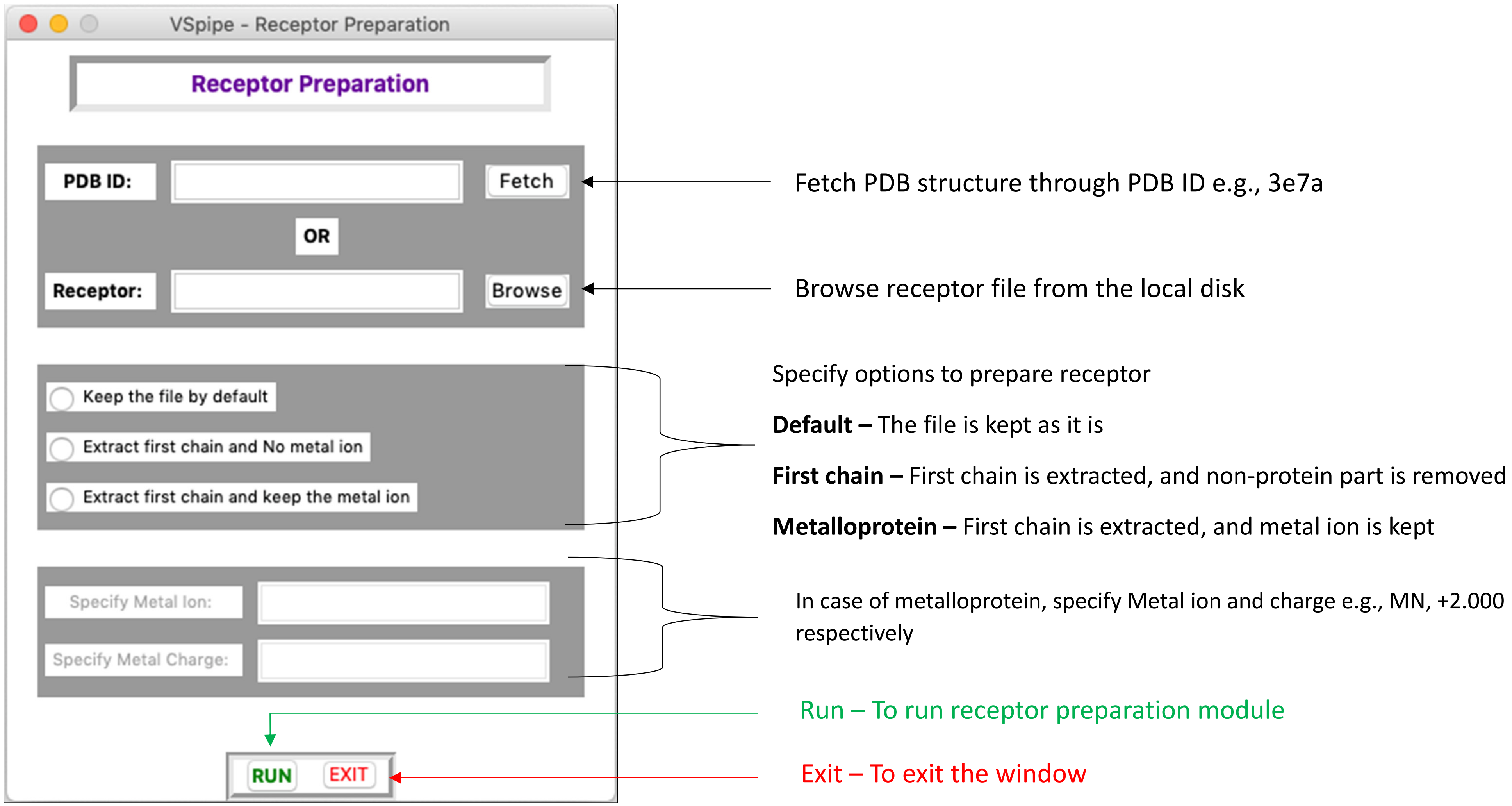

Figure S3. Receptor Preparation GUI Window

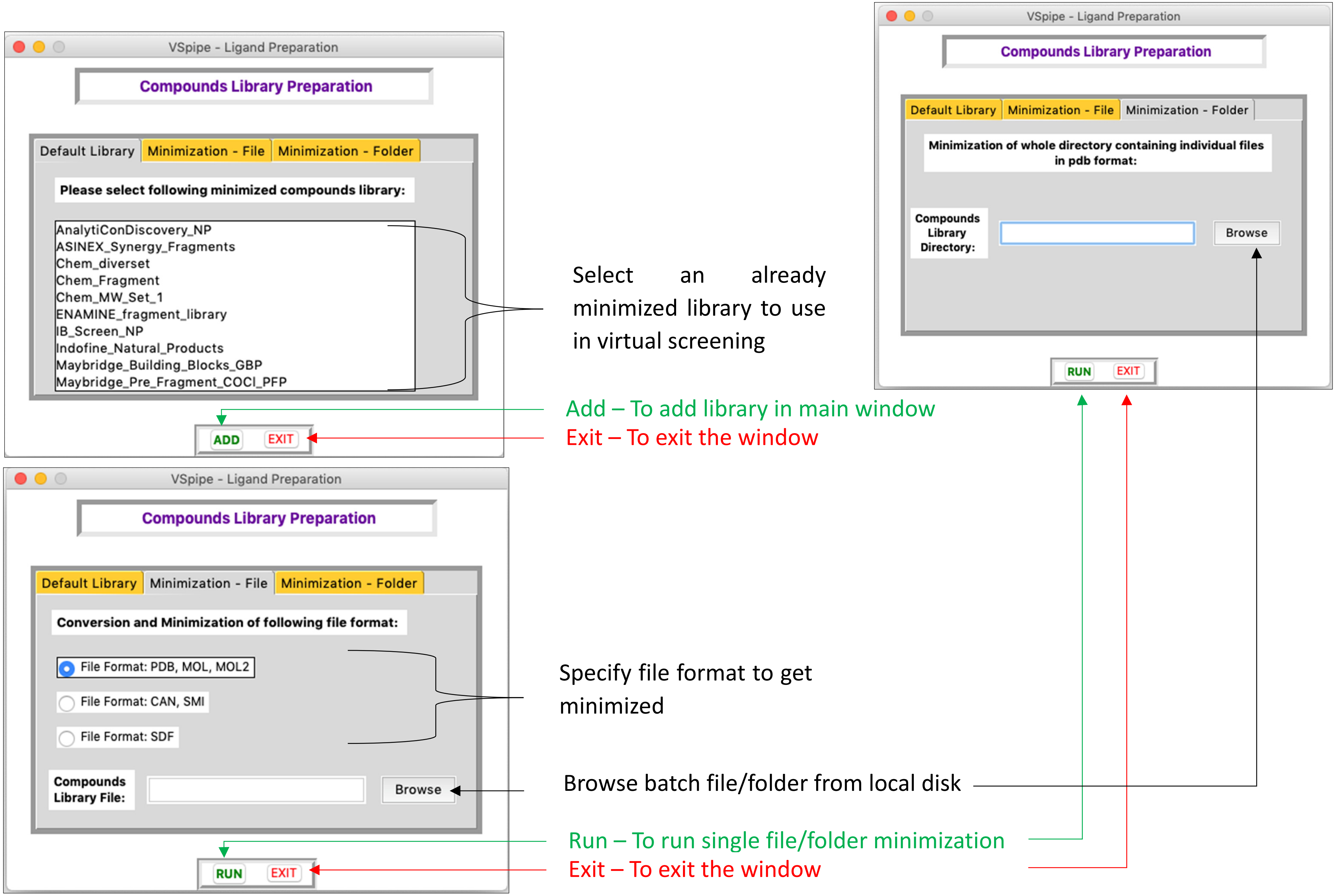

Figure S4. Compounds Library Preparation GUI Window

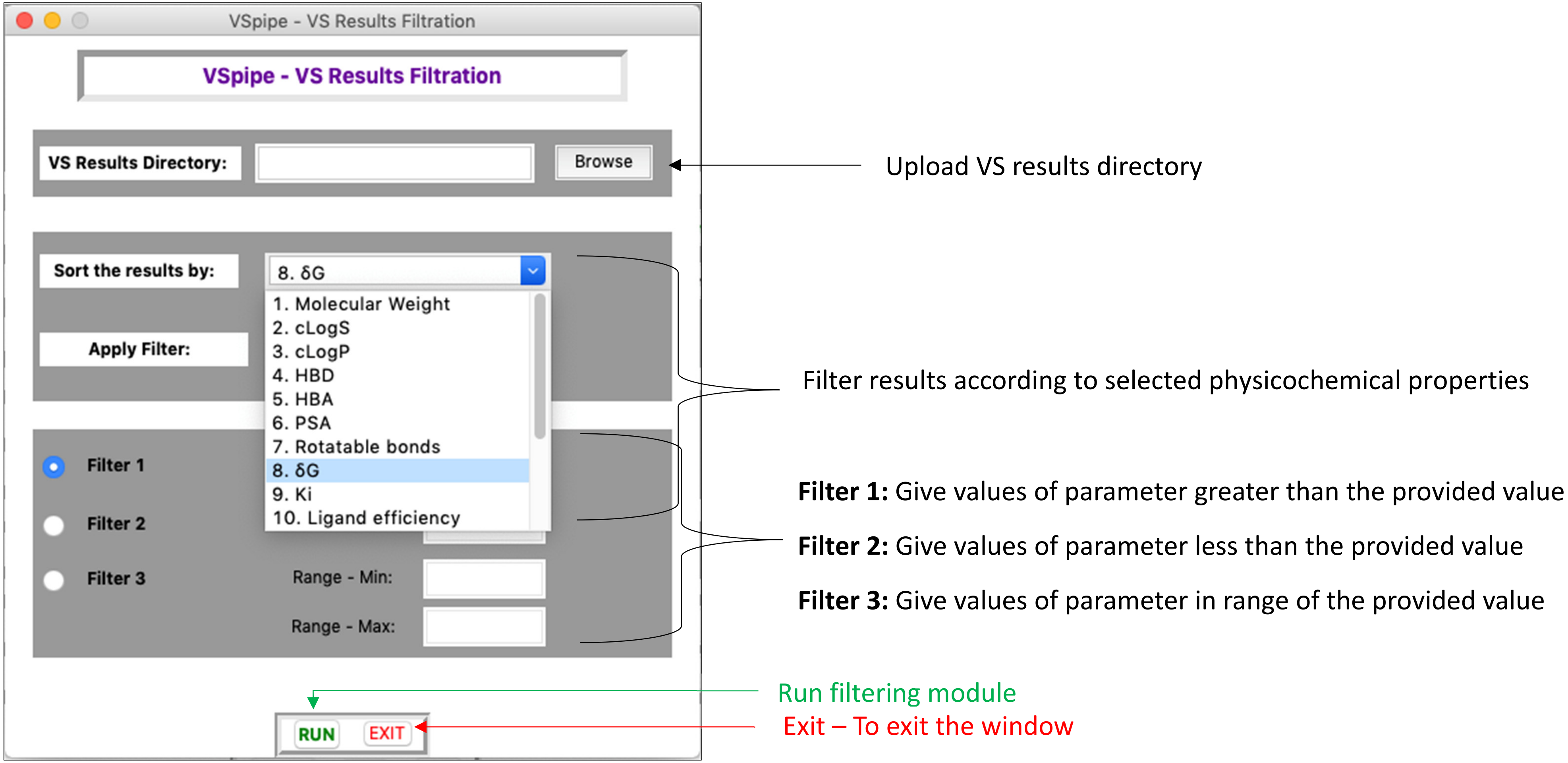

Figure S5. Filtration GUI Window for Filtering Screening Results \

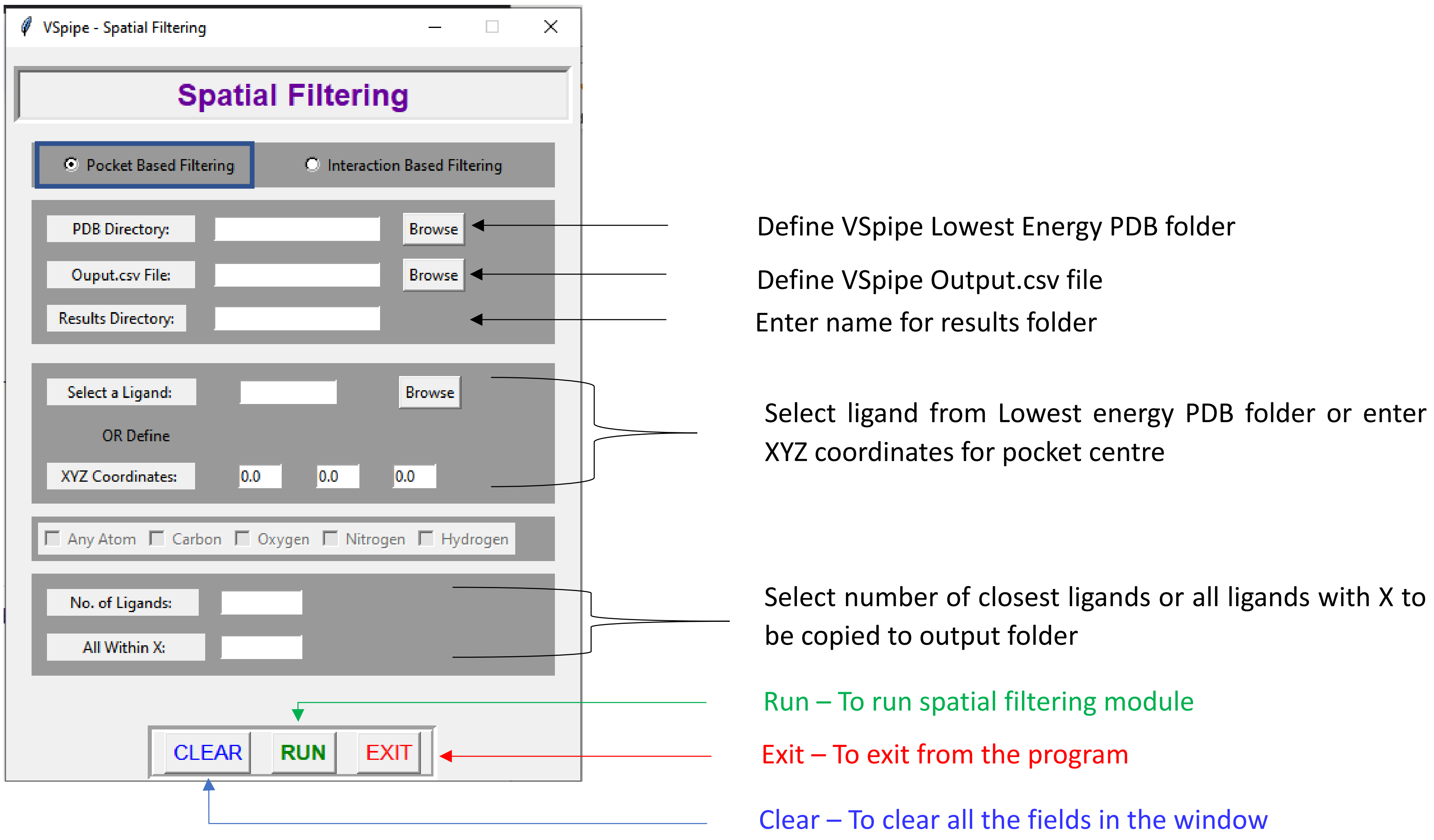

Figure S6. Spatial Filtering GUI window – Pocket-based Filtering

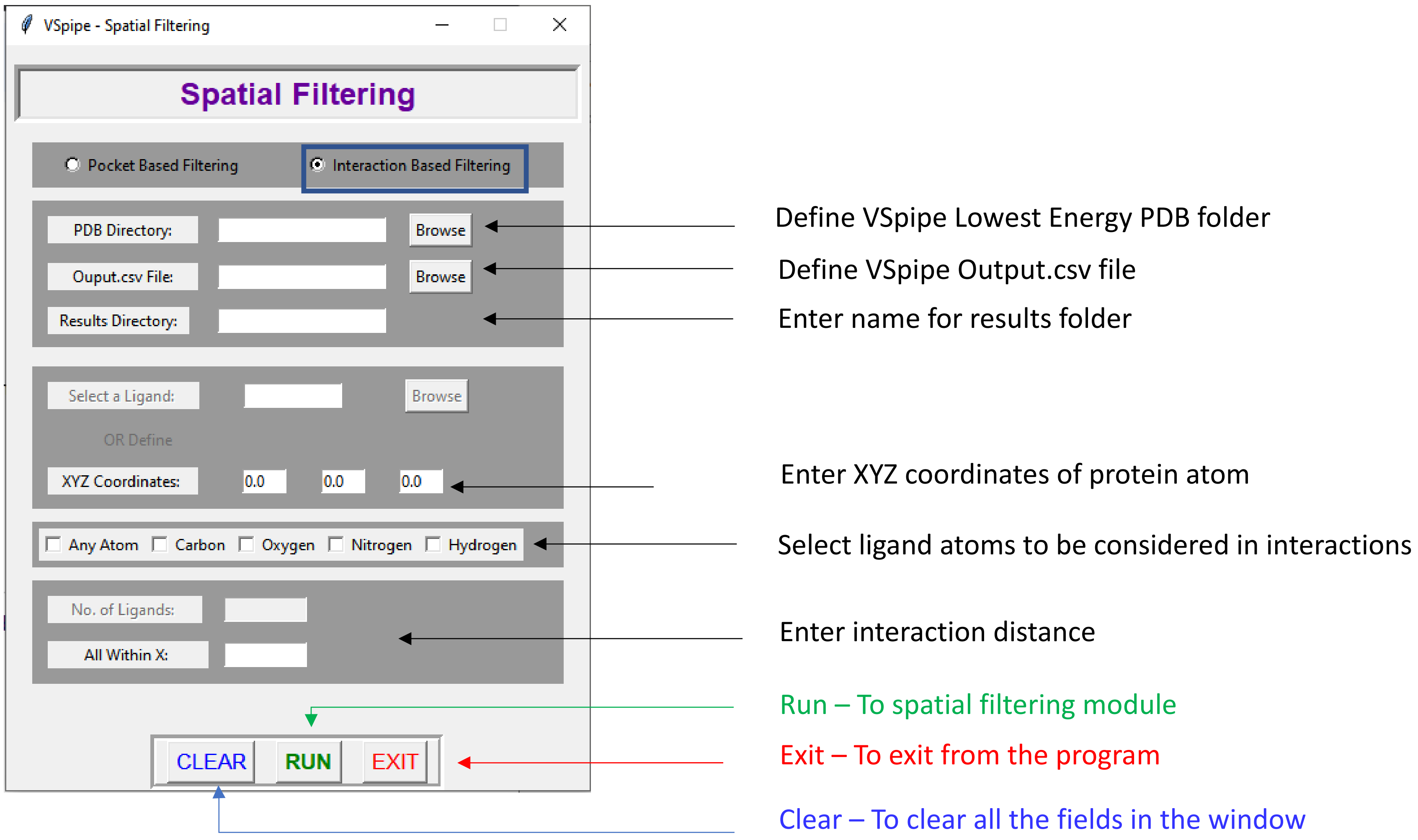

Figure S7. Spatial Filtering GUI window – Interaction-based Filtering.

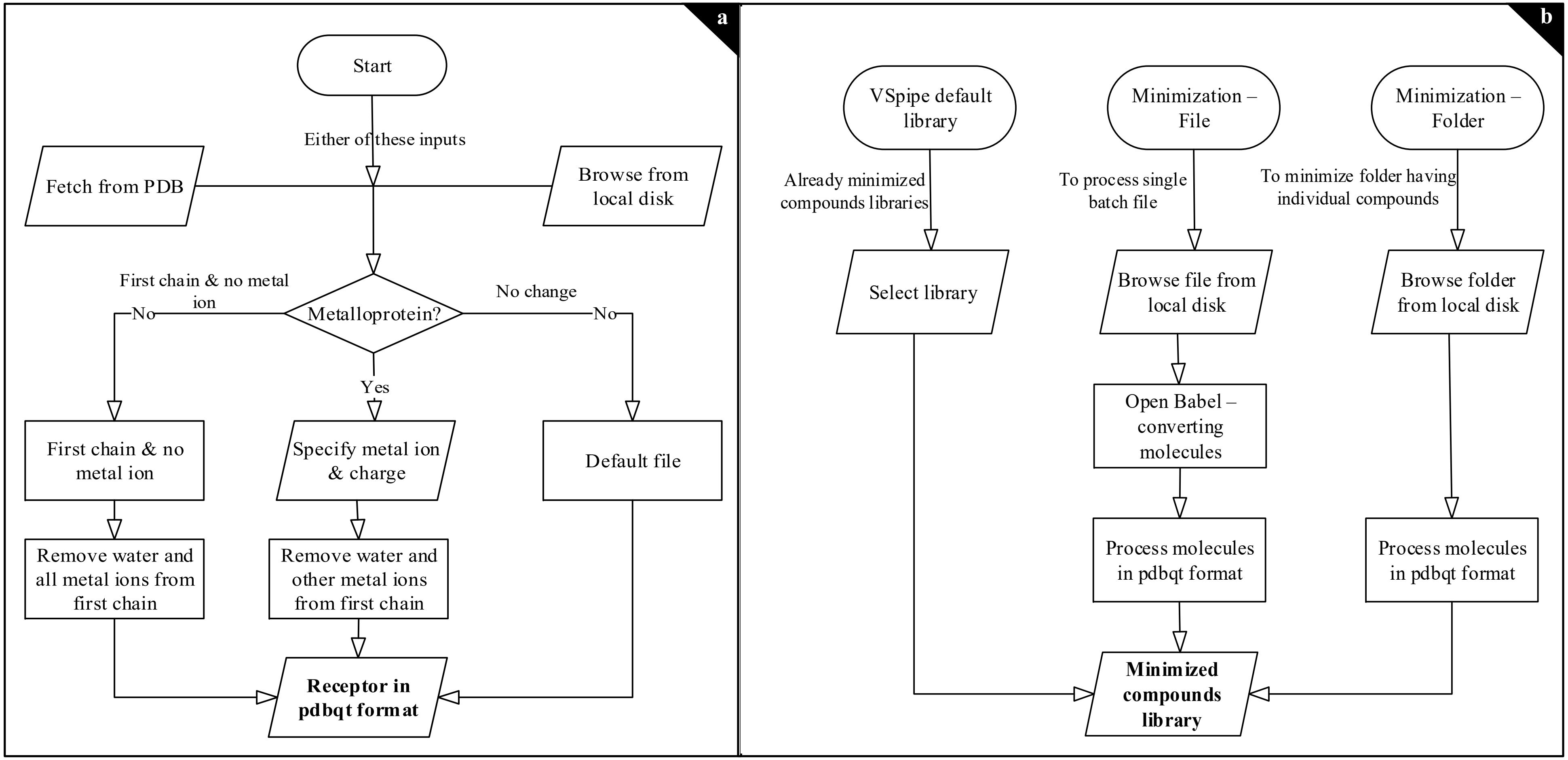

Figure S8. a) Receptor Preparation: module that processes the target protein as the receptor onto which the ligands will be later docked. b) Compounds Library Preparation: module used to prepare the ligands to be docked onto the receptor in which users can either select one of the already minimized libraries included in VSpipe-GUI, select one of their already minimized libraries, or minimize an external library if provided in PDB, mol2, or SDF format.

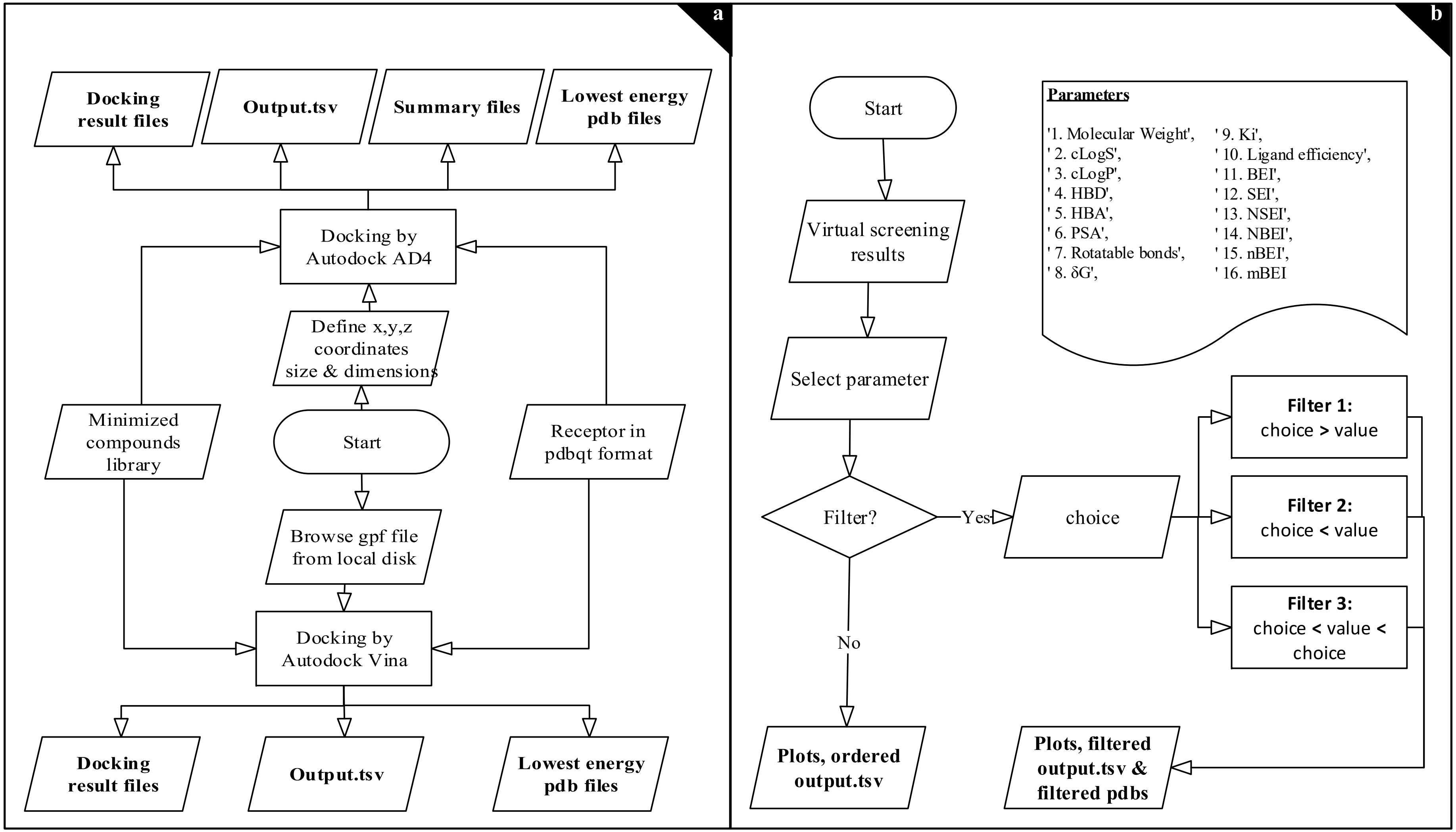

Figure S9. a) Docking: this module requires the 3D coordinates that define the grid box in which the docking will take place, which can be provided by either entering the coordinate values (i.e., values for coordinates x, y, and z) on VSpipe-GUI or by uploading the GPF file on the tool (the coordinates will be extracted from this file). In addition, users need to select the software that will be used for the docking, either AD4 or Vina. b) Filtering: this module allows for the docking results to be filtered according to a given threshold by the user for one of the 16 physicochemical properties the screened ligands have had calculated during the virtual screening.
